# A randomized digital behavioral intervention for prenatal and postpartum weight outcomes in women with overweight or obesity: the GROWell trial

**DOI:** 10.1186/s12884-026-08846-3

**Published:** 2026-02-26

**Authors:** Leigh Ann Simmons, Jennifer E. Phipps, Sebastian Castro-Alvarez, Paige Smith, Courtney Overstreet, Alina Patrikeyeva, Paige Gilliland, Victoria F. Keeton, Devon Noonan

**Affiliations:** 1 Betty Irene Moore School of Nursing, University of California, Davis, 2570 48th St, Sacramento, CA 95817 USA; 2 Department of Human Ecology , University of California, Davis, 1 Shields Ave, Davis, CA 95616 USA; 3 Department of Obstetrics and Gynecology, UC Davis Health, 4860 Y St #2500, Sacramento, CA 95817 USA; 4 Duke University School of Nursing, 307 Trent Dr, NC 27710 Durham, USA

**Keywords:** Overweight, Obesity, Mobile health, Diet quality, Pregnancy, Postpartum, Gestational weight gain, Postpartum weight retention, Randomized clinical trial

## Abstract

**Background:**

Rising rates of overweight and obesity globally have led to an increasing number of women who enter pregnancy with excess weight, posing significant health risks to mothers and infants. Mobile health interventions, such as smartphone apps, may be a solution to improving pregnancy outcomes, however, limited randomized studies have examined this approach for gestational weight gain (GWG) and postpartum weight retention (PPWR). We report results from a double-blinded, randomized control trial of Goals for Reaching Optimal Wellness (*GROWell*), a mobile app and text-based intervention designed to improve diet quality and associated weight outcomes in pregnant and postpartum women.

**Methods:**

Women living in California with BMI = 25–42 kg/m^2^ and a singleton, uncomplicated pregnancy were recruited via social media or clinic in early pregnancy from January 2021 through March 2023. After completing a baseline survey, participants were randomized to *GROWell* or an educational control. Participants completed online surveys of diet quality and other health behaviors and self-weighed using study-provided Bluetooth scales. Modified Poisson regression tested for differences in excess GWG and 6-month PPWR.

**Results:**

Using block randomization in permuted blocks of three based on prepregnancy BMI, race/ethnicity, and recruitment source) 453 racially and ethnically diverse participants (237 attention control, 216 intervention) were enrolled. Mean age was 33.6 ± 4.1 years and mean BMI was 30.9 ± 4.28 kg/m^2^. Excess GWG was observed in 36% of the control group and 35% of the intervention group. PPWR was observed in 26% of the control group and 23% of the intervention group.

**Conclusions:**

Compared to an attention control, *GROWell* was not associated with lower rates of excess GWG or PPWR. However, study rates of excess GWG and PPWR were lower in both groups compared to averages in the US and several Western nations. Future studies should investigate the potential of text-based educational support on weight and other health indicators among childbearing women.

**Trial registration:**

ClinicalTrials.gov identifier NCT04449432. Registered on June 26, 2020.

**Supplementary Information:**

The online version contains supplementary material available at 10.1186/s12884-026-08846-3.

## Introduction

Rising rates of overweight and obesity in the United States and other high- and middle- income countries across the globe have led to an increasing number of women entering pregnancy with excess weight, posing significant health risks to both mother and child [1–6]. Maternal overweight and obesity are associated with greater gestational weight gain (GWG) and postpartum weight retention (PPWR) [7, 8]. The Institute of Medicine defines excessive GWG as more than 25 pounds for women who begin pregnancy with overweight BMI, and more than 20 pounds for those with obesity. Excessive GWG is linked to an array of pregnancy complications, including placental abnormalities, blood clots, preeclampsia, gestational diabetes, and suboptimal infant health indicators like low Apgar scores [9–11]. Moreover, women who gain excess gestational weight are more likely to retain excessive weight postpartum, which frequently results in beginning their subsequent pregnancies at a higher BMI [12]. This establishes a cycle of excessive GWG and PPWR that contributes to long-term health risks such as cardiovascular disease and cancer as women age [12–14].

Excess GWG is particularly common among women with pre-pregnancy overweight or obesity [8] and is generally more harmful than inadequate weight gain [12]. Data from the US showed that 64% of women who entered pregnancy with an overweight BMI and 58.7% of those who entered pregnancy with an obese BMI experienced excessive GWG [15]. Globally, these rates range from approximately 18% to over 60%, with higher rates reported in high-income countries and among women with higher pre-pregnancy BMI [16, 17]. While women who experience excess GWG are more likely to have PPWR than those who gain within IOM guidelines [12], most women in the U.S. and globally do not return to their prepregnancy weight within the first six months postpartum [18–21], a timeframe that has been shown to increase risk for never returning to prepregnancy weight [22]. Multiple factors, including mood, breastfeeding practices, diet quality, physical activity, prepregnancy BMI, race, ethnicity, age, parity, smoking status, education, and income, are all associated with excess GWG and PPWR [4, 8, 23–25].

Numerous interventions have been developed to promote healthier pregnancy weight gain and facilitate postpartum weight loss [26, 27]. Most interventions for pregnant and postpartum women are still recruited for and delivered in-person [28]. Mobile and telehealth interventions along with social media, online, and community-based recruitment are becoming more common, especially since the COVID-19 pandemic. These remote approaches offer an opportunity to reach a broader and more diverse population, including women in rural or underserved areas who may have limited access to academic health centers, which often serve as hubs for research trials [29]. The *Goals for Reaching Optimal Wellness (GROWell)* intervention was primarily informed by self-regulation theory, operationalized through self-monitoring (weekly weight reporting and goal adherence via Bluetooth scales and text messaging) and diet-focused goal setting (two tailored, incremental dietary quality goals supported with daily texts) [30]. These strategies also align with social cognitive theory constructs such as self-efficacy and behavioral control, providing a strong theoretical basis for the intervention [31]. The objective of this study was to investigate whether a randomized controlled trial of GROWell, a mobile health intervention offering personalized dietary guidance, could reduce GWG and PPWR among pregnant women in California with prepregnancy overweight or obesity, compared to an attention control group receiving personalized information on pregnancy and early infancy, but not diet or infant feeding.

## Methods

### Participants

A detailed study protocol was published previously [30]. Briefly, we recruited women 18–44 years old with prepregnancy BMI of 25–42 who were 10–16 weeks’ gestation of a confirmed singleton pregnancy and nulliparous or > 12 months since a previous birth. We excluded women who had known pregnancy complications (e.g., preexisting hypertension or diabetes, tobacco use). Based on preliminary work, a power analysis was performed to determine that with a sample size of 354, we could have 80% power to detect differences in excess GWG and PPWR between intervention and control arms [30].

### Recruitment

Due to the launch of the trial coinciding with the COVID-19 pandemic shutdown in March 2020, we primarily recruited participants via social media. We partnered with StudyPages, by Yuzu Labs PBC, to run our social media ad campaign and conduct initial screening and consent. We also conducted limited clinic-based recruitment in a large urban academic medical center. All participants consented electronically using an IRB-approved form, which was administered virtually on their mobile device or a clinic-provided mobile device.

### Randomization and treatment assignment

Treatment assignment occurred after completion of the baseline questionnaire. The randomization sequence was computer-generated by a third-party vendor (Pattern Health), who also managed allocation. Computerized stratified randomization with permuted blocks of size 3 were used to assign eligible participants to *GROWell* or the attention control with an equal allocation ratio. Prepregnancy BMI categories, race/ethnicity, and recruitment method (clinic or via social media) were the a priori stratification variables. Researchers and participants were both blinded to group assignment. Pattern Health blinded research staff to group assignment using numeric codes. When study staff abstracted electronic health record data, they did not have information on what group the participant identifier indicated. While participants knew the content of their messaging, they were unaware whether their assignment represented the intervention or control.

### Protocol adherence

The only deviation from the published protocol was that we used Poisson regression, rather than logistic regression, after determining that Poisson regression provides a more appropriate estimate of risk ratios in randomized control trials. Otherwise, no changes to methods or outcomes were made after trial commencement. All analyses followed the intention-to-treat principle, meaning that participants were analyzed in the groups to which they were randomized, regardless of adherence, withdrawal, or loss to follow-up.

### Study procedures

Additional details regarding procedures can be found in the previously published protocol [30]. In brief, participants were followed at 26–28 and 36–38 weeks prenatally, and 3 and 6 months postpartum. They completed online questionnaires and provided weights via study-provided Bluetooth scale. Increasing remuneration at each timepoint was utilized to encourage retention. Labor and delivery information was obtained via medical record abstraction. Breastfeeding status was assessed via text messaging upon hospital discharge and weekly thereafter until self-reported termination of breastfeeding or study completion.

### Intervention design

**Fig. 1:**
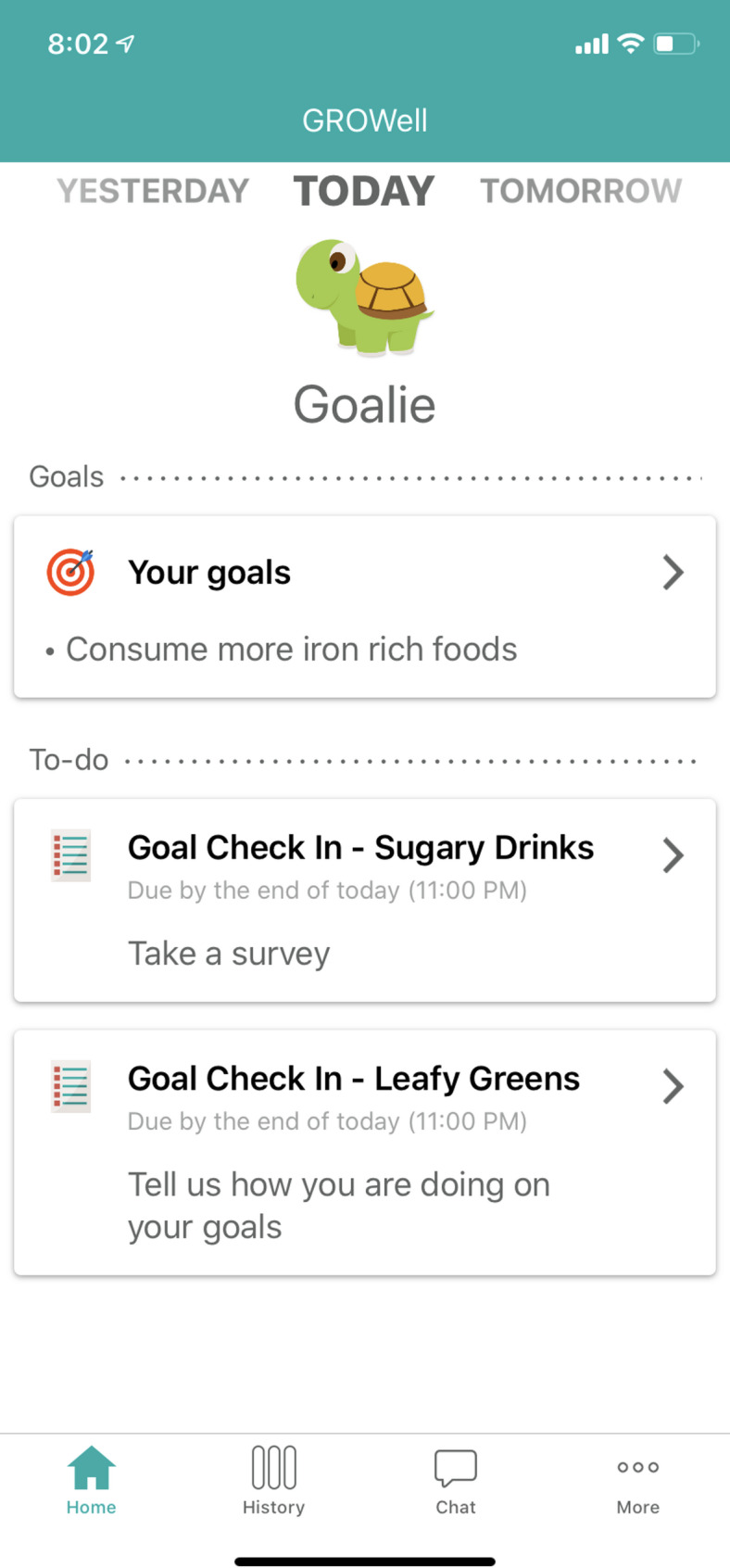
Example screenshot of the GROWell interface showing the goal navigation menu

Upon enrollment, participants took a brief online survey on their specific dietary behaviors, their readiness and self-efficacy to change these behaviors, dietary preferences and restrictions, and whether they preferred to receive and respond to messages via text message or directly through the GROWell app. The GROWell platform delivered identical content through both the mobile app and text messages with all responses and weight data flowing into the same behind-the-scenes system. The background system handled the personalization, data integration, and goal tracking, while the app served as a convenient hub where participants could view and manage this information. Text messages offered an alternative user-facing channel, allowing participants to complete all study activities entirely by text without losing any functionality if users preferred. Based on survey responses, the app’s prescription algorithm assigned two personally tailored dietary change goals (see Fig. 1). The algorithm prioritized goals in highest need of change and for which the participant had high self-efficacy and readiness. One goal was a “do” goal (e.g., eat more leafy greens, increase folic acid containing foods), and one goal was a “don’t” goal (e.g., avoid high sodium foods, reduce fast food consumption).

During the prenatal period, the library of dietary behavioral goals (see Table 1) was consistent with controlling energy intake and meeting nutrition recommendations for pregnancy. During the postpartum period, goals were assigned to consider breastfeeding status and to promote safe postpartum weight loss (0.5-2 pounds per week). In addition to goal assignment, the app integrated several supportive features, including a study-provided Bluetooth scale for remote weight tracking, automated feedback messages, and adherence monitoring through weekly self-reports and a text-back protocol. The automated diet algorithm incorporated participants’ dietary preferences, such as a nut-free, red meat-free, vegetarian, vegan or gluten-free diet. Text messages offered meal suggestions, cooking strategies, educational content, and problem-solving tips specifically tailored to the participant’s previous week’s progress on their goals. These features supported engagement, self-monitoring, and adaptive goal adjustments, all with the goal to increase self-efficacy for improved diet quality.

**Table 1:** *GROWell* dietary goals and sample messaging

Foods/Nutrients to reduce:	Sample messaging	Foods/Nutrients to increase:	Sample messaging
Fried foods	“Try steaming your veggies tonight instead of frying.”	3 fruits daily	“Grab a piece of fresh fruit when you’re craving a snack.”
Sugary drinks	“Switch things up with flavored seltzer water.”	Whole grains every day	“Replace white rice with brown rice or quinoa for extra fiber.”
Salty snacks	“Curb your craving and get an extra kick of fiber with a couple whole grain crackers. Top with a little nut butter, guacamole, salsa, or hummus.”	Healthy fats every day	“Add a tablespoon of flaxseed to oatmeal or a smoothie for a healthy breakfast.”
High sugar sweets	“Added sugar should be no more than 10% of your diet. Baked apples with cinnamon can satisfy your sweet tooth.”	Lean protein daily	“Plain Greek yogurt with berries is a great protein packed breakfast.”
High fat/high salt seasoning	“Choose air-popped popcorn instead of oil-popped for a great snack with less saturated fat.”	Healthy breakfast daily	“Have lunch for breakfast - Top whole wheat toast with a smear of avocado, cherry tomatoes, 1/2 cup of arugula, and a drizzle of balsamic vinegar.”
Fast food	“Pre-make healthy snacks for the end of the day or your ride home. This will help you avoid fast food restaurants.”	Iron rich foods	“Sprinkle black beans onto your salads for a boost of iron.”
Salt	“When eating restaurant food, request that your meal be served with sauces and condiments on the side or served without extra added salt.”	Dark leafy greens daily	“Add some freshness and texture to your wraps and sandwiches by adding extra greens.”
Red meat	“Red meat is a good source of iron, which is really important in the 3rd trimester. But it’s also high in fats, so make it a weekly treat.”	3 veggies daily	“Grated zucchini is easy to add to sauces, pizza, soups and stir-frys. Try adding this versatile summer squash to your every day recipes to increase your veggie consumption.”
Processed meats	“Try nitrate-free bacon instead of regular, if you really need bacon with your breakfast (and keep it to once a week or less).”	Healthy calcium daily	“Try a fruit sorbet for dessert instead of ice cream! Add poppy seeds or chia seeds to boost the calcium.”
		Folate-rich foods	“Asparagus is rich in folate. Simply sautee in a skillet and top with a squeeze of lemon and some black pepper.”
		Fiber-rich foods	“Green peas are high in fiber. Incorporate some into your next meal.”

Participants virtually engaged with *GROWell* Monday through Friday. Once weekly, participants reported via text or the mobile application how many days in the previous week they adhered to their goals. On the other four days, participants received supportive, goal-focused text messages with problem-solving skills and dietary tips tailored to the previous week’s progress on their goal. When participants did not respond, we used a text-back protocol that automatically reminded them to report adherence and maintain engagement. As women successfully met their goals with > 70% adherence (i.e., at least 5 out of 7 days) for 2 consecutive weeks, they were assigned the next goal for which they had the highest need combined with the highest readiness to change.

### Personalization algorithm

**Fig. 2:**
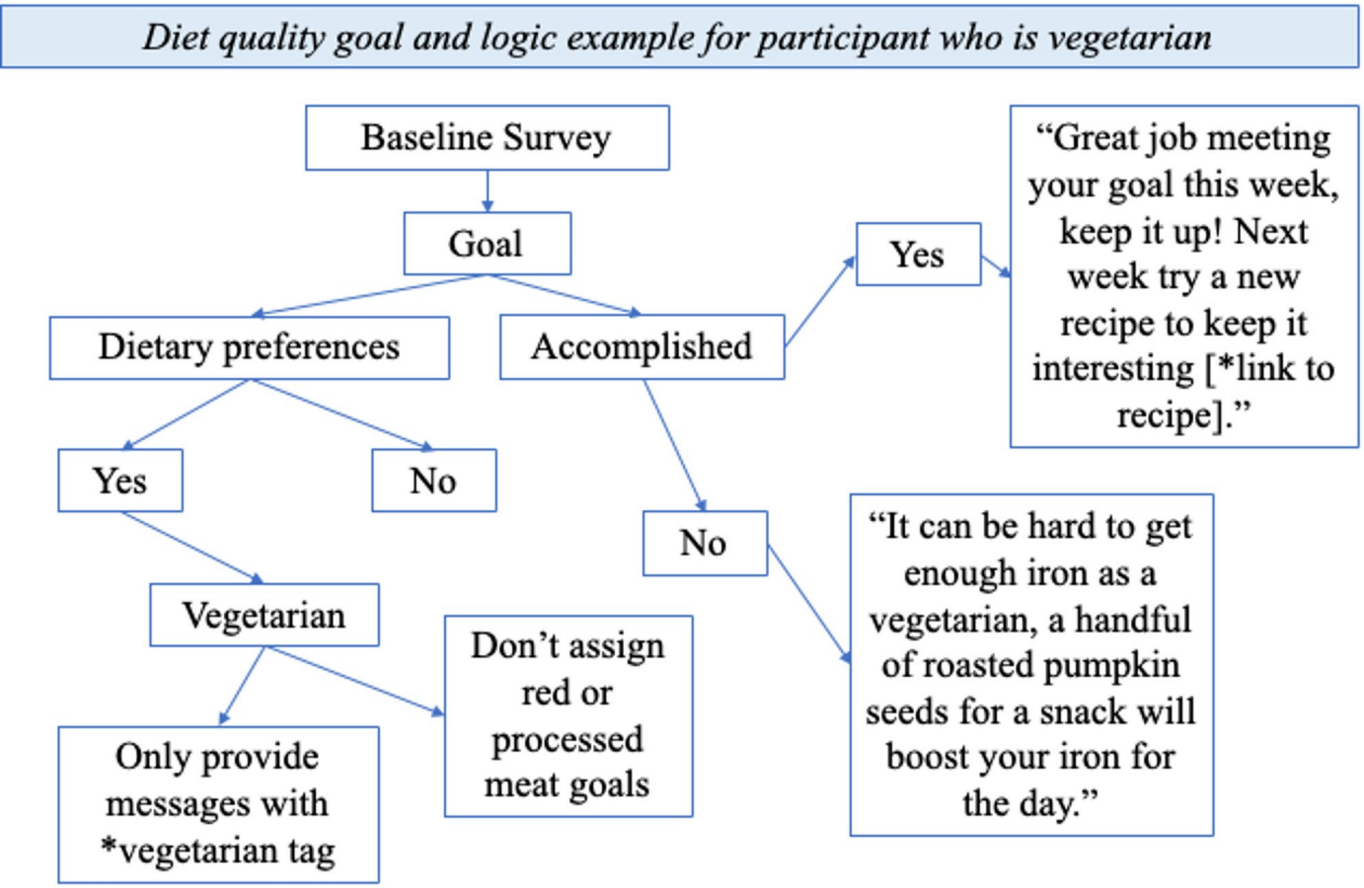
Logic flow of the GROWell algorithm for an example participant

All messaging for both the intervention and control included tags related to aspects of personalization that participants entered in their surveys. Tags included dietary preferences: no red meat, no eggs, no gluten, no nuts, no meat, no dairy, breastfeeding status, whether the participant lived alone or with a partner, whether they were going back to work after their baby was born, and whether they had other children at home. Then goal messaging was updated based on the tags. For instance, if a postpartum participant in the intervention group was a vegetarian, allergic to nuts, and breastfeeding, she would not receive a goal about red meat and would not receive messaging or recipes with meat or nuts. Her goals in the postpartum period would also consider the additional nutrient needs of breastfeeding. Alternatively, a pregnant participant in the control arm who was planning to return to work and not planning to breastfeed would receive messaging about planning for childcare. See Fig. 2 for an example and demonstration of the prescription algorithm logic.

### Attention control

We isolated the effects of diet-focused goal setting, self-monitoring, and feedback in *GROWell* by providing attention control participants with information that was specific to pregnancy, labor, delivery, and early infancy, purposefully avoiding content focused on maternal or infant nutrition or diet (Table 2). Based on the information we collected for personalization (described above), participants assigned to the attention control received: (a) pregnancy, fetal development, and labor and delivery information provided via text once weekly between enrollment and 37 weeks’ gestation; and (b) education on infant development via weekly texts from birth to 6 months postpartum.

**Table 2:** Attention control educational topics and sample messaging

Prenatal	Sample messaging	Postpartum	Sample messaging
Pregnancy	“You may not be showing yet, but have you thought about how to share the news? https://www.thebump.com/a/pregnancy-announcement-ideas”	Infant development	“After birth a baby only sees black, white, and shades of gray. Pictures with geometric patterns help babies to develop focus. Around 4 months old baby starts to develop color vision.”
Fetal development	“Your baby can hear your voice! The more you talk to your baby, the better language skills your baby will have. Starting this week spend 10 min every day talking or reading to your baby.”	Parenting	“Babies love the sound of your voice. Also, studies show that the more words babies hear, the better their later reading, writing, and speaking skills are. Read, sing, and talk to them as much as you can!”
Labor and delivery	“If you haven’t taken a hospital tour yet, make sure to sign up. This way you and your labor support person know where you’re going on the big day. Ask your OBGYN about how to sign up for a tour at your hospital.”	Relationships	“Try to schedule an hour each week to be with your partner alone without baby- even if it is just while baby is asleep in the other room. This will keep you connected as you integrate this new child into your family.”
Early infancy education	“Your baby will be here soon! In your baby’s first weeks, you can support healthy emotional and social development. Hold, kiss, rock, touch, talk to, and sing to your baby often. When you are feeding your baby, keep eye contact. Try not to multitask on your phone or with other distractions.”	Sleep	“The American Pediatric Association recommends babies sleep on their backs for all sleep times- naps and at night- until baby is able to roll by [him/herself] confidently from tummy to back and from back to tummy.”
Social connection	“Social support is important for good emotional health. Make sure you have 1 or 2 people you can count on for help if you need it. This is also a good time to seek out new moms’ groups in your area.”	Mood	“Talk to your doctor or midwife if you are feeling overwhelmed with sadness or worry. The “baby blues” happen to most women, but they should resolve within 2 weeks of birth and you should not feel like you cannot take care of yourself or baby.”
Relationships	“Adding a baby to your family affects your partner relationship. These are common issues that come up: couple time, parenting styles, household chores, sex, grandparents’ role, and money. Pick one issue to discuss each week for the next 6 weeks.”	Medical guidelines for mom & baby	“Keep up with your and your baby’s postpartum doctor visits. See the following link for recommended vaccine timelines from the American Academy of Pediatrics: https://publications.aap.org/redbook/resources/15585/”
Sleep	“It may start to become more challenging to get good sleep. Follow these tips to help you sleep better at night. https://www.whattoexpect.com/pregnancy/sleep-solutions/pregnancy-sleep/”	Infant crying	“For most babies, crying peaks around 6 weeks and then gradually gets better. If crying does not improve, take baby to his/her provider.”
Safety concerns	“As your baby and belly grow, you might notice that you lose your balance when walking. Wear flat or low-heeled shoes to lower the risk of falling.”	Adjusting family to new baby	“Schedule some time alone with your older children to help them adjust to life with the new baby. Try just 15 min a day of reading a story and cuddling while baby is in someone else’s arms or asleep.”
Mental health	“As your due date gets closer, it is normal to feel anxious about the birth of your baby. Start now finding ways to create calm in your life. Add 1 of these easy breathing practices to your day: http://www.drweil.com/drw/u/ART00521/three-breathing-exercises.html	Returning to work	“It can be really stressful to go back to work after having a new baby. Make sure to plan in advance for this transition. Give yourself time to build trust in your childcare situation and the way life will function without you home full-time.”

### Measures

#### GWG

GWG (in kg) was computed by taking the difference between the weight at 36–38 weeks of pregnancy and the weight at baseline. We categorized this variable into excessive or non-excessive based on IOM recommendations for prepregnancy BMI to create a binary variable excess *GWG*. Participants who started the study with overweight were considered to have excess GWG if they gained more than 11.34 kg (25lbs) and participants who started the study with obesity were considered to have excess GWG if they gained more than 9.07 kg (20lbs).

#### PPWR

PPWR (in kg) was computed by taking the difference between the weight at 6 months postpartum and the weight at baseline. Then, we categorized participants into experiencing PPWR or not. Participants for whom their weight at 6 months postpartum was *≥* 5% of their weight at baseline were considered to have PPWR.

#### Diet quality

To assess diet quality, participants completed the Rapid Eating and Activity Assessment for Patients (REAP) [32] at baseline, 26–28 weeks, 36–38 weeks, 3 months postpartum, and 6 months postpartum. This questionnaire is a valid and reliable measure of diet quality and consists of 24 items scored from 1 to 3, with total scores varying from 24 to 72. Higher scores indicate better diet quality. The Cronbach’s alpha for this scale was 0.73.

#### Physical activity

The Pregnancy Physical Activity Questionnaire (PPAQ) was used to measure physical activity [33]. We computed the total score based on 14 items with total scores ranging between 0 and 844.4. Larger values indicate greater physical activity in metabolic equivalents. The Cronbach’s alpha for the PPAQ was estimated at 0.71.

#### Depression

The Edinburgh Postnatal Depression Scale (EPDS) was used to measure depression [34]. The scale consists of 10 items scored from 0 to 3 and total scores can range from 0 to 30, with larger scores indicating more clinically significant depressive symptoms. Cronbach’s alpha for this scale was 0.83.

#### Covariates

Age, BMI, race and ethnicity, mother’s education, and parity at baseline. We combined race and ethnicity into one variable to reduce the presence of missing data. Hence, the category Hispanic includes all participants who identified as Hispanic independent of the race they reported. Other variables of interest included whether the participants had adverse pregnancy outcomes (such as hypertensive disorders of pregnancy, gestational diabetes mellitus, and preterm birth), the total number of times they stepped on the Bluetooth scale during the study, and whether they were exclusively breastfeeding at 6 months postpartum.

#### Statistical analysis

Analyses were conducted in R (4.4.2). We used descriptive statistics to summarize baseline characteristics and conducted independent samples t-tests or chi-square (χ^2^) tests to examine differences in covariates of interest between the intervention and control groups. Modified Poisson regression models [35, 36] assessed intervention effects on the primary outcomes: *excess GWG and 6-month PPWR*. Poisson regression was chosen because it directly estimates risk ratios, which are more appropriate and interpretable than odds ratios in RCTs. Model I included only group assignment. Model II additionally adjusted for covariates: age, race/ethnicity, education, parity, BMI, diet quality, physical activity, depression, adverse pregnancy outcomes, and Bluetooth scale use. Exclusive breastfeeding status at 6 months postpartum was also adjusted for in the PPWR model. Missing data were addressed using multiple imputation [37] for both primary outcomes (EGWG and PPWR) and covariates, generating 30 imputed datasets. Multiple imputation assumes data are missing at random, meaning that the probability of missingness can be explained by observed variables. To make this assumption more plausible, we included observed covariates predictive of missingness (e.g., group assignment, baseline EPDS scores, race/ethnicity) in the imputation models. This approach is widely used in clinical trials to minimize bias and preserve statistical power. Sensitivity analyses using complete-case (listwise deletion) data were also conducted, and results were nearly identical, indicating robustness of the findings. Herein, results from multiple imputation models are presented. Significance was defined as p *≤* 0.05 with 95% confidence intervals.

## Results

### Recruitment, retention, and adherence

**Fig. 3:**
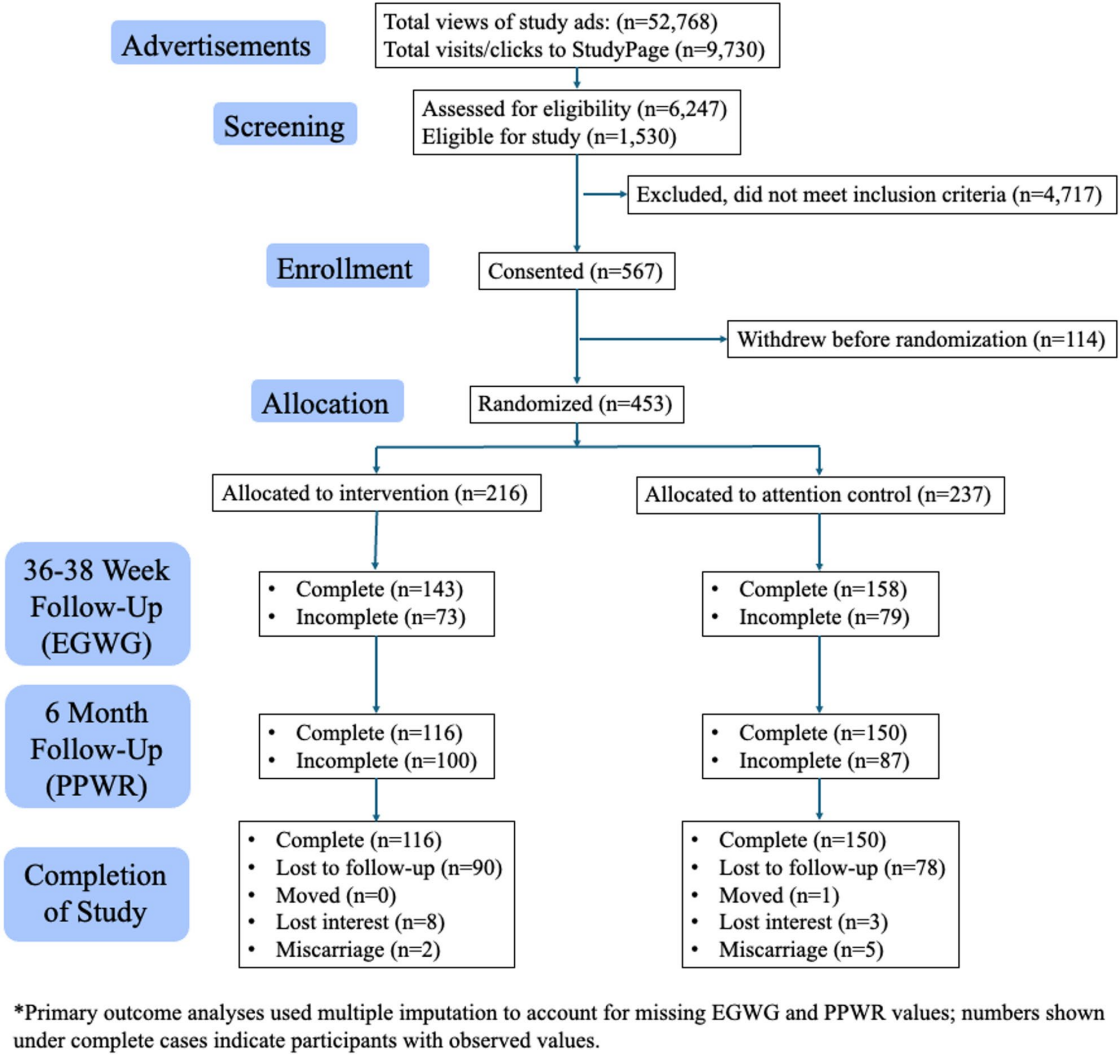
GROWell CONSORT diagram

Figure 3 presents the CONSORT diagram for *GROWell*. Recruitment took place from January 2021 to March 2023 and was closed after 567 participants consented. Of those, 114 withdrew before randomization, leaving 453 participants who were randomized: 216 to the intervention group and 237 to the control group. Follow-up concluded in February 2024, when the final participant completed their 6-month postpartum surveys. By the end of the trial, 268 participants had completed the 6-month follow-up: 117 in the intervention group and 151 in the control group. Reasons for attrition included: loss to follow-up, relocation, loss of interest, or pregnancy loss (see Fig. 3). Among those assigned to the intervention, adherence to weekly text-based dietary goal tracking averaged 72% over the course of the study.

### Participant demographics and health characteristics

Tables 3 and 4 present the descriptive characteristics of the sample at baseline compared by experimental group assignment. There were no significant differences between groups, indicating that the randomization worked well. However, the average number of times participants stepped on the scale is larger for participants in the intervention group.

**Table 3:** Baseline descriptive statistics for continuous variables by experimental group

	Control			Intervention			t-test	
	Mean	SD	*N* Missing	Mean	SD	*N* Missing	t	*p*-value
**Age**	33.4	4.30	0	33.8	3.86	0	-1.03	0.30
**BMI**	31.3	4.28	5	31.6	4.55	3	-0.72	0.47
**REAP**	53.0	6.03	0	52.7	6.58	0	0.42	0.67
**PPAQ**	135.7	81.51	0	130.8	74.31	0	0.67	0.51
**EPDS**	6.6	4.06	0	7.0	4.18	0	-0.93	0.35
**Total Weights**	10.2	13.59	0	15.3	19.66	0	-3.18	< 0.01

*SD* Standard deviation, *REAP *Rapid Eating and Activity Assessment for Patients, *PPAQ* Pregnancy Physical Activity Questionnaire, *EPDS* Edinburgh Postnatal Depression Scale.

**Table 4:** Frequency tables for covariates by experimental group

		Control	Intervention	χ^2^ test	
				χ^2^	p-value
*Race/Ethnicity (self-reported)*	White	124	119	1.22	0.88
	Black	10	8		
	Asian	29	25		
	Hispanic	63	50		
	Multi-racial/Other	11	13		
	Unknown	0	1		
*Mother’s highest education completed*	Post-Baccalaureate	171	146	0.78	0.38
	Some College or Less	66	69		
	Unknown	0	1		
*Primigravida*	Yes	103	91	0.04	0.85
	No	134	125		
	Unknown	0	0		
*Experienced an adverse pregnancy outcome*	Yes	64	54	0.004	0.95
	No	138	121		
	Unknown	35	41		
*Exclusive breastfeeding at 6 months postpartum*	Yes	63	54	0.36	0.55
	No	88	63		
	Unknown	86	99		

We were able to determine whether participants had excess GWG for 301 participants (*n* = 143 in the intervention group and *n* = 158 in the attention control group). Excess GWG occurred in 50 intervention participants (35%) and 57 attention control participants (36%). Similarly, PPWR could be assessed for 266 participants (*n* = 116 in the intervention group and *n* = 150 in the control group). PPWR was observed in 27 intervention participants (23%) and 39 attention control participants (26%).

### Excess GWG

Table 5 shows the results of the modified Poisson regression models based on multiple imputation using excess GWG as the dependent variable. The estimated risk ratio is presented with its corresponding lower and upper bounds of the 95% confidence intervals. Model I, which includes only group assignment, indicates that the intervention did not have a significant effect on excess GWG. Model II, which adjusts for all covariates of interest, yielded similar results. Some of the covariates had a significant effect on the outcome. For example, “Asian” participants were less likely (RR = 0.478 [0.273-0.837]) to gain excessive gestational weight compared to “White” participants.

**Table 5:** Modified poisson regression for EGWG with multiple imputation

	Model I			Model II		
	Est. RR	LB	UB	Est. RR	LB	UB
Intercept	0.392***	0.322	0.476	3.200	0.507	20.181
Group						
Control	Ref.	Ref.	Ref.	Ref.	Ref.	Ref.
Intervention	1.011	0.777	1.315	1.049	0.803	1.37
Covariates						
Age				0.983	0.95	1.018
Race/Ethnicity:						
White				Ref.	Ref.	Ref,
Black				0.638	0.249	1.636
Asian				0.478*	0.273	0.837
Hispanic				0.812	0.562	1.172
Multi-racial/Other				0.852	0.475	1.528
Mother’s Education:						
Post Baccalaureate				Ref.	Ref.	Ref.
Some College or Less				1.214	0.91	1.619
Primigravida:						
Yes				Ref.	Ref.	Ref.
No				0.921	0.69	1.23
Baseline BMI				0.975	0.946	1.006
Baseline REAP score				0.995	0.975	1.015
Baseline total PPAQ score				0.997+	0.995	1
Baseline EPDS score				1.010	0.977	1.043
Adverse pregnancy outcome:						
No				Ref.	Ref.	Ref.
Yes				0.949	0.716	1.259
Number of weight measurements				0.988	0.976	1.001
Num.Obs.	453			453		
Num.Imp.	30			30		

*Est*. *RR* Estimated risk ratio, *LB* Lower bound of the 95% confidence interval, *UB* Upper bound of the 95% confidence interval, *Ref.* Reference category, *REAP* Rapid Eating and Activity Assessment for Patients, *PPAQ* Pregnancy Physical Activity Questionnaire, *EPDS* Edinburgh Postnatal Depression Scale.

+ *p* < 0.1, * *p* < 0.05, ** *p* < 0.01, *** *p* < 0.001

### PPWR

Table 6 presents the results of the modified Poisson regression models using multiple imputation with PPWR as the dependent variable. The intervention did not have a significant effect on PPWR in either model. In Model II, which includes all covariates of interest, none of the covariates were significantly associated with PPWR.

**Table 6:** Modified poisson regression for PPWR with multiple imputation

	Model I			Model II		
	Est. RR	LB	UP	Est. RR	LB	UP
Intercept	0.335***	0.261	0.428	0.760	0.052	11.027
Group						
Control	Ref.	Ref.	Ref.	Ref.	Ref.	Ref.
Intervention	0.952	0.667	1.359	0.996	0.701	1.416
Covariates						
Age				1.015	0.969	1.065
Race/Ethnicity:						
White				Ref.	Ref.	Ref.
Black				0.997	0.388	2.562
Asian				0.792	0.457	1.371
Hispanic				0.813	0.521	1.271
Multi-racial/Other				1.291	0.564	2.956
Mother’s Education:						
Post Baccalaureate				Ref.	Ref.	Ref.
Some College or Less				1.161	0.789	1.71
Primigravida:						
No				Ref.	Ref.	Ref.
Yes				1.393	0.92	2.11
Baseline BMI				0.950+	0.907	0.995
Baseline REAP score				0.999	0.973	1.026
Baseline total PPAQ score				1.002	0.999	1.005
Baseline EPDS score				1.004	0.961	1.05
Adverse pregnancy outcome:						
No				Ref.	Ref.	Ref.
Yes				0.942	0.616	1.441
Number of weight measurements				0.993	0.981	1.005
Exclusive breastfeeding at 6 months postpartum:						
Yes				Ref.	Ref.	Ref.
No				0.844	0.573	1.243
Num.Obs.	453			453		
Num.Imp.	30			30		

Est. *RR* Estimated risk ratio, *LB* Lower bound of the 95% confidence interval, *UB* Upper bound of the 95% confidence interval, Ref.: Reference category; *REAP* Rapid Eating and Activity Assessment for Patients, *PPAQ* Pregnancy Physical Activity Questionnaire, *EPDS* Edinburgh Postnatal Depression Scale.

+ *p* < 0.1, * *p* < 0.05, ** *p* < 0.01, *** *p* < 0.001

## Discussion

While rates of excessive GWG and PPWR did not differ significantly between the GROWell intervention and control groups, both groups demonstrated lower rates of these outcomes compared to national and international averages, after adjusting for relevant covariates. In the US, an estimated 55–61% of women with pre-pregnancy BMIs greater than 25 kg/m^2^ experience excessive GWG [38]. Rates in some Western European countries (e.g., United Kingdom, Sweden, and Germany) range from 40 to 51% [10, 16]. In contrast, only 35.5% of participants in our cohort experienced excessive GWG. Estimates of PPWR vary across studies, but up to 75% of women may retain weight at 1 year postpartum [18, 22]. In our cohort, however, only 32.0% of participants had not returned to their pre-pregnancy BMI by 6 months postpartum. These relatively low rates may reflect several factors rather than the intervention alone. One factor may be the educational level of participants, which was relatively high at 69.9% with a Bachelor’s degree compared to the US average of 41.7% [39]. However, studies of Western European women do not consistently show that higher educational levels are linked with lower rates of excess GWG [10, 40]. Another factor may be motivation and self-selection into the study. Thirdly, participants in both the experimental and control groups had access to a Bluetooth scale and could self-weigh at any time. Self-weighing has been shown to be useful in weight loss maintenance [41–43] and may have contributed to the observed outcomes. A fourth contributor may be that our attention control provided a sense of support to participants, especially as they navigated pregnancy and a new baby, some during the height of the COVID-19 pandemic. Some studies suggest that lack of social support is associated with excess GWG [44, 45]. It may be that a simple, text-based informational intervention could influence weight outcomes. Future studies should investigate mechanisms of action for behavioral interventions aimed at pregnant and postpartum women, including consideration of multiple behavior interventions. Motivation to change behavior, self-weighing, and remote delivery of education could be considered alone or in combination as a strategy for achieving recommended GWG and postpartum weight loss, especially among women who have limited access to care, such as those living in rural areas.

It is important to note that we did not observe improvements in diet quality, which was assessed using the validated REAP survey focusing on overall dietary patterns rather than specific nutrient intake. One reason for this may be that we focused on the prenatal period as opposed to the preconception period. Some evidence suggests that if dietary changes can be implemented preconception, the impact on GWG and PPWR may be stronger [46]. Future studies should investigate whether the *GROWell* intervention implemented prior to pregnancy may have more of an impact on GWG and PPWR, as well as other important maternal and infant outcomes, such as adverse pregnancy outcomes. Moreover, our intervention aimed to improve diet quality over a relatively short period and during pregnancy, a time when eating patterns and food cravings often fluctuate unpredictably [47, 48]. Previous studies have linked common pregnancy cravings for high calorie, energy-dense foods (e.g., sweets, breads, salty snacks, fast food) to excess GWG [49]. Future dietary studies should consider including craving management, such as through mindful eating, as a behavioral target.

Interventions combining diet quality improvements with increased physical activity have shown the greatest success for reducing GWG and PPWR [50–54], yet the results across studies remain mixed. While we did not attempt to increase physical activity in this trial, we did assess and control for self-reported intensity of physical activity and did not find that it influenced our primary outcomes. Additionally, *GROWell* appears not to have impacted rates of adverse pregnancy outcomes, though this needs to be tested in a larger cohort. Many prior interventions that focused on reducing GWG and PPWR have not examined potential health impacts, such as on rates of adverse pregnancy outcomes, infant health indicators, or maternal cardiovascular metrics like blood pressure and biomarkers (e.g. cholesterol, hemoglobin A1c). Consequently, the potential benefits for overall maternal and child health of interventions focused on GWG or PPWR remain unclear and require further investigation.

A strength of *GROWell* was our diverse cohort. In particular, 24.9% identified as LatinX, which is the largest growing ethnic group in the US [55], thus increasing the generalizability of our results for this group. While our *GROWell* intervention was available in Spanish, only 1 participant used the Spanish version of the app. Future work should explore how to culturally tailor *GROWell* to the LatinX community beyond the simple translation of the English version of the app that we provided. Our app also did not assess for experiences of racism and other social or structural determinants of health, which have known impacts on GWG and PPWR [56, 57]. 

*GROWell* recruitment began in January 2021 and ended in March 2023. During this period, the impact of COVID-19 on prenatal and postpartum care drastically changed from limiting in-person care availability and causing anxiety for pregnant women and their families to becoming more of a routine illness comparable to the flu [58]. Our studies have shown that the pandemic influenced health behaviors including diet, physical activity, and virus-protection measures (e.g. mask wearing, hand washing) [59–61]. We cannot estimate the impact of the pandemic on who enrolled, who completed the study, and how they adhered to dietary goals. Arguably, the pandemic had such a significant effect on individual health behaviors, access to food, options for exercise, and care seeking, all of which impact weight, that the pandemic alone may have been the reason for null results.

Our study also demonstrated geographic diversity based on regions of California as well as urban, suburban, and rural communities. Likely this is because we pivoted to remote recruitment when the pandemic shut down in-person research and clinical care [62]. This is a strength of our study, because most prenatal and postpartum behavioral interventions recruit in-person. With the use of geographic targeting, we were able to increase the reach of our social media ads in zip code regions with high percentages of residents considered historically and presently marginalized. These strategies should be amplified in future clinical trials to decentralize participation and increase representation of groups often not included in scientific research.

Additional strengths of our study relate to strategies we employed to reduce bias and increase access. First, we used a double blinded randomized trial design, thus increasing the rigor and reducing the effect of potential confounders on results. Second, due to our text-back protocols, we had excellent adherence to goals and follow-up compared to similar mobile health studies [53, 63]. Third, the remote trial design allowed for decentralization of study locations, increasing reach for women who did not live near our academic medical center. Fourth, we used a Bluetooth scale rather than relying on self-reported weights, allowing for accurate measurement. Lastly, participants were able to select whether they wanted to receive SMS text messages or use a mobile application to access the intervention, which allowed women without reliable internet access to participate. All these aspects of the GROWell trial increased the inclusive nature of our behavioral intervention with robust outcome data.

## Limitations

Our study also has limitations. First, not all participants completed data collection at each time point for all surveys or weight measurements. Second, we relied on self-report measures of physical activity and sleep rather than having participants wear activity trackers, and thus our ability to control for these important confounders may have been limited by reporting bias. Third, while the racial and ethnic backgrounds of our cohort were highly diverse, most were highly educated and all sought out this research opportunity. These self-selection factors reflect different phenotypes of behavioral intervention responders and may bias our results. Fourth, we used participant baseline weight as a proxy for prepregnancy weight, which may vary slightly but is generally considered a valid measure [64, 65]. Additionally, the Spanish version of the intervention was not available immediately and once it was available, initially the default language for the mobile app was the same as the default language of the participant’s phone, even if that was English. Fifth, common pregnancy symptoms such as nausea and vomiting, particularly in the first trimester, can affect diet quality, potentially influencing GWG and PPWR, and we did not account for these in our models. Lastly, although we utilized an evidence-based algorithm to assign goals that consider participant readiness to change specific behaviors, dietary messaging was personalized to the goal, not the person (e.g., no specific cultural foods). Further, we focused on diet quality, not intake. This approach could have contributed to null results. Future versions of the app could consider more culturally congruent foods as well as an approach where participants select goals from a recommended list that the algorithm produces.

## Conclusion

While this mobile health dietary intervention did not reduce excess GWG or PPWR compared to the attention control, participants in both arms gained less weight during pregnancy and retained less postpartum weight than national and some global averages. We were able to conduct an inclusive, rigorous trial with a diverse cohort. Future studies should investigate the impact of *GROWell* delivered prior to pregnancy, improve reach to individuals who have lower educational levels, culturally tailor content and goals, and target multiple behaviors, such as physical activity and diet combined.

## Supplementary Information

Below is the link to the electronic supplementary material

Supplementary Material 1

## Data Availability

The datasets created and analyzed during the current study are available from the corresponding author on reasonable request.
