# Supplementary material for: A randomized digital behavioral intervention for prenatal and postpartum weight outcomes in women with overweight or obesity: the GROWell trial
Source: BMC Pregnancy Childbirth. 2026 Feb 26;26:368. doi: 10.1186/s12884-026-08846-3 (PMC13041289; doi:10.1186/s12884-026-08846-3)
Supplement: Supplementary file 1 — Supplementary Material 1 [file 12884_2026_8846_MOESM1_ESM.pdf]

GROWell Baseline Demographic Questions:

Please enter your date of birth:

Enter date

What is your race?

White

Black or African American

Asian

Native American or Alaska Native

Native Hawaiian or Pacific Islander

More than one race

Unknown or Not Reported

Are you Hispanic?

Yes/No

If yes, what is your Hispanic origin?

Mexican

Cuban

Puerto Rican

US American

Other Hispanic

Unknown

What is your gender?

Female

Non-binary

Transman

Transwoman

Prefer not to answer

Other

What is your street address?

Enter address

What is your city?

Enter city

What is your state?

Enter state

What is your zip code?

Enter date

What is your current height?

Enter height

What is your current weight?

Enter weight

Your marital status:

Single

Married

Divorced

Widowed

Separated

Prefer not to answer

Your education:

Elementary/Middle School

Some High School

High School

Some College

Post-baccalaureate

Unknown

Not including you, how many are in your household under 18?

0

1

2

3

4

5

6

7

8

9

10

11

12 or more

Including you, how many are in your household over 18?

1

2

3

4

5

6

7

8

9

10

11

12 or more

Do you live with:

Partner

Mother/Mother figure

Father/Father figure

Grandmother

Grandfather

Sister

Brother

Other adult relative

Other unrelated adult

Your children

Other children

Live alone

Prefer not to answer

How long have you lived at your current residence (in years)?

Less than 1 year

1

2

3

4

5

6

7

8

9

10

11

12 or more years

What is your occupation?

Type in occupation

Are you an essential employee?

Yes/no

What best describes your occupation?

Health care provider

Health care administrator

Cleaning service in health care

Food service in health care

General food service

Food delivery service

General delivery service

Utility worker

Other

Eating habits questionnaire

How many days per week do you drink sugary drinks?

0-1

2-3

4-5

6-7

Are you ready to drink fewer sugary drinks next week? (y/n)

How many days per week do you eat sweets?

0-1

2-3

4-5

6-7

Are you ready to eat fewer sweets next week? (y/n)

How many days per week do you eat whole grains?

0-1

2-3

4-5

6-7

Are you ready to eat more whole-grains next week? (y/n)

How many days per week do you eat fast food?

0-1

2-3

4-5

6-7

Are you ready to eat less fast food next week? (y/n)

How many days per week do you eat fried foods?

0-1

2-3

4-5

6-7

Are you ready to eat less fried food next week? (y/n)

How many days each week are you conscious of your sodium intake?

0-1

2-3

4-5

6-7

Are you ready to be more mindful of your sodium levels starting next week? (y/n)

How many days per week do you add seasonings and condiments without much nutritional value?

0-1

2-3

4-5

6-7

Are you ready to try to use more healthful seasonings or condiments next week? (y/n)

How many days per week do you eat breakfast?

0-1

2-3

4-5

6-7

Are you ready to eat breakfast more days next week? (y/n)

How many days per week do you eat healthy fats?

0-1

2-3

4-5

6-7

Are you ready to eat more healthy fats next week? (y/n)

How many days per week do you eat iron-rich foods?

0-1

2-3

4-5

6-7

Are you ready to eat more iron-rich foods next week? (y/n)

How many days per week do you eat at least 4 fruits?

0-1

2-3

4-5

6-7

Are you ready to eat more fruits next week? (y/n)

How many days per week do you eat at least 4 vegetables?

0-1

2-3

4-5

6-7

Are you ready to eat more vegetables next week? (y/n)

How many days per week do you eat folate-rich foods?

0-1

2-3

4-5

6-7

Are you ready to eat more folate-rich foods next week? (y/n)

How many days per week do you eat dark, leafy greens?

0-1

2-3

4-5

6-7

Are you ready to eat more dark, leafy greens next week? (y/n)

How many days per week do you eat fiber-rich foods?

0-1

2-3

4-5

6-7

Are you ready to eat more fiber-rich foods next week? (y/n)

How many days per week do you eat salty snacks?

0-1

2-3

4-5

6-7

Are you ready to eat less salty snacks next week? (y/n)

How many days per week do you eat red meat?

0-1

2-3

4-5

6-7

Are you ready to eat less red meat next week? (y/n)

How many days per week do eat processed meats?

- 0-1
- 2-3
- 4-5
- 6-7

Are you ready to eat processed meat less often next week? (y/n)

How many days per week do get adequate calcium?

- 0-1
- 2-3
- 4-5
- 6-7

Are you ready to eat more calcium-rich foods next week? (y/n)

How many days per week do you eat lean protein?

- 0-1
- 2-3
- 4-5
- 6-7

Are you ready to eat more lean protein next week? (y/n)

Do you avoid or have an allergy to any of the following foods?

- Dairy
- Eggs
- Fish
- Gluten
- Nuts
- Red Meat
- White Meat
- None of the above

Almost done! Click next to submit your answers
